# Identification of Occupational Cancer Risks in British Columbia, Canada: A Population-Based Case—Control Study of 1,155 Cases of Colon Cancer

**DOI:** 10.3390/ijerph8103821

**Published:** 2011-09-26

**Authors:** Raymond Fang, Nhu Le, Pierre Band

**Affiliations:** 1 American Academy of Physician Assistants, Suite 1300, 2318 Mill Road, Alexandria, VA 22314, USA; 2 Cancer Control Research, British Columbia Cancer Agency, 675 West 10th Avenue, Vancouver, BC V5Z 1L3, Canada; E-Mails: nle@bccrc.ca (N.L.); pierre_band@hc-sc.gc.ca (P.B.); 3 Health Canada, 50 Columbine Drive, Tunney’s Pasture, Ottawa, ON K1A 0K9, Canada

**Keywords:** occupational cancer, colon cancer, cancer risk, occupational exposure, carcinogen

## Abstract

**Objective:**

Cancer has been recognized to have environmental origin, but occupational cancer risk studies have not been fully documented. The objective of this paper was to identify occupations and industries with elevated colon cancer risk based on lifetime occupational histories collected from 15,463 incident cancer cases.

**Method:**

A group matched case-control design was used. All cases were diagnosed with histologically proven colon cancers, with cancer controls being all other cancer sites, excluding rectum, lung and unknown primary, diagnosed at the same period of time from the British Columbia Cancer Registry. Data analyses were done on all 597 Canadian standard occupation titles and 1,104 standard industry titles using conditional logistic regression for matched data sets and the likelihood ratio test.

**Results:**

Excess colon cancer risks was observed in a number of occupations and industries, particularly those with low physical activity and those involving exposure to asbestos, wood dusts, engine exhaust and diesel engine emissions, and ammonia.

**Discussion:**

The results of our study are in line with those from the literature and further suggest that exposure to wood dusts and to ammonia may carry an increased occupational risk of colon cancer.

## 1. Introduction

Colon cancer is one of the leading cancer causes of death. In Canada, colon cancer is the third most common form of cancer (after prostate cancer and lung cancer in men and breast cancer and lung cancer in women) and the third leading cancer cause of death (after lung cancer and prostate cancer in men and lung cancer and breast cancer in women) [1].

Despite the common occurrence of colon cancer, its etiology is not well established; age, family history [2], lifestyle factors [3–6] including daily alcohol use, low-fiber diets, a high consumption of animal fat and red meat, physical inactivity [7–10], obesity [11–13] and smoking [14] are reported risk factors. The evidence regarding occupational exposures related to the occurrence of colon cancer is generally limited and/or not consistent [15]. Exposures to asbestos [16,17], dyes in textile industry [18], hydrazine in rocket fuels [19], pesticides in agriculture [20], engine exhaust and diesel engine emissions [21] and occupations as managers [22], male farmers [23], painters, police, guards and firemen, motor transport workers and clerks as well as exposures in non-metallic mineral products, leather goods, meat, poultry, and fish products industries [24] have been reported to be associated with a higher risk of colon cancer. A consistent relationship, however, has primarily been found for physically inactive occupations [25–29].

We have, as part of a program aimed at detecting occupational risk factors in British Columbia, Canada, collected lifetime occupational histories from 15,463 incident cancer cases, of which 1,155 had a diagnosis of colon cancer. Occupational risks for this group of patients are presented in this article by assessing risk difference in comparison with internal controls consisting of all other cancer patients excluding lung and rectum cancer and cancer of unknown primary sites.

## 2. Materials and Methods

### 2.1. General Methodology

The general methodology of the study has been described in detail in a previous article [30]. Briefly, male cancer patients aged 20 years and older ascertained by the population-based British Columbia Cancer Registry (BCCR) for the years 1983 to 1990 inclusively, received a self-administered questionnaire requesting lifetime job descriptions, occupation and industry titles, and duration and period of work, as well as information on ethnic origin, education, and lifetime smoking habits. Questions about lifetime consumption of alcoholic beverages, which were initially omitted, were added to the questionnaire during the first year of the study. In the event of a patient’s death, information was requested from the spouse or closest living relative. For the first two years, questionnaires were sent to all cases. Data collection continued until 1,000 completed questionnaires were accrued for each tumor site, or until 31 December 1990, whichever occurred first. The distribution of patients’ usual occupations and usual industries as well as assessments of response bias and of questionnaire validation and reliability was conducted and previously reported [30]. The anatomic site and pathology of the primary tumor were coded using the 9th revision of the International Classification of Diseases [31] and the International Classification of Diseases for Oncology [32], respectively. Occupations and industries were coded according to the Canadian Standard Occupational Classification (SOC) and the Canadian Standard Industrial Classification (SIC), respectively [33,34]. In the SOC, occupations are coded according to two-digit major group codes, three-digit minor group codes, and four-digit unit group codes. In the SIC, industries are coded according to two-digit major group codes, three-digit group codes, and four-digit class codes. During the data-collection period, questionnaires were sent to all 25,726 eligible male cancer cases ascertained by the BCCR and 15,463 (60.1%) were returned. Histological confirmation of diagnosis was obtained in all cases. For colon cancer, questionnaires were sent to 1,768 eligible cases, of which 1,156 (65.4%) were returned.

### 2.2. Statistical Methodology

A group matched case-control design was used. Cases comprised all 1,156 colon cancer patients. Controls comprised internal controls with all other cancer sites, excluding lung cancer (n = 2,998), rectum cancer (n = 1,095) and cancers of unknown primary site (n = 708), matched to the cases on exact age and year of diagnosis. These were based on a few methodological considerations. First, using other cancers as controls has a few advantages over population controls with regard to recall and interviewer bias when using general population controls, a mixture of live and deceased controls can be expected when selecting other cancer patients as controls and there are logistical and cost advantages in using other cancer patients as controls. Thus the study design using cancers as controls is likely to be used increasingly in the future, particularly in cancer registry settings [35]. Next, since lung cancer is mainly caused by smoking, excluding it from the control group would avoid potential bias of having too many smokers in controls. In addition, matching on age and year of diagnosis is a way to control for major confounders. Including year of diagnosis is a control of time when rapid changes in population structure were happened from 1960s through 1990s.

Data were analyzed using conditional logistic regression for matched sets and the likelihood ratio test [36,37] in a two-step procedure. In step 1, the effect of the following non-occupational confounding variables was assessed: marital status; education (less than 8 years; 8 to 11 years; high school; post-secondary); smoking (starting age at smoking, average number of cigarettes, pipe or cigars smoked per day, total years smoked); alcohol consumption (starting age at consuming alcohol; average of number of bottles of beer, glasses of wine, ounces of spirits consumed per day; total years of consuming alcohol); and person who filled out the questionnaire (self or proxy). Variables were selected in a forward fashion each being examined separately; Potentially important confounders (*P* < 0.2) were then included in the model and the remaining ones were examined; the process was repeated until no factor with *P* < 0.2 was identified. In step 2, each occupation and industry was assessed separately using conditional logistic regression in which all significant confounding variables identified in step 1 were taken into account. The cut point of 0.20 was chosen in step 1 to ensure that the process has sufficient power to identify potentially important confounders.

Matched case-control analyses were carried out using PECAN software [38], test of significance of the adjusted odds ratios (ORs), p-values and 95% confidence intervals were calculated and presented. Analyses were performed for the following two different estimates of occupational exposures: ever occupation/industry (whether a job was ever held in a given occupation/industry under consideration) *versus* never, and usual occupation/industry (job with the longest held lifetime employment in a given occupation/industry). People in either ever or usual occupation/industry categories were always compared to their counterparts who were never in such occupation/industry. Due to all 597 occupation titles and 1,104 industry titles were analyzed and small numbers of cases in many occupation/industry categories, we intended to maximally present results of the analyses in this article with occupations and industries as long as their number of cases is five and over.

## 3. Results

Matching resulted in 1,155 colon cancer cases having at least one matching control and 7,552 controls, leaving an overall control to case ratio of 6.5:1. Site distribution of controls is listed in Table 1.

The characteristics of cases and controls are shown in Table 2.

Statistically significant ORs were noted for the following variables: smoking duration and education level as shown in Table 3; the latter was mainly due to the unknown category.

Odds ratios by ever and usual occupation and industry categories are shown in Tables 4 and 5, respectively. In the ever occupation/industry categories and for most major groups, a number of occupations/industries had significantly elevated ORs. In many instances, the corresponding ORs in the usual occupations/industries categories were either non-significant or had fewer than five cases. Concordance for statistically significantly increased ORs between ever and usual categories at least at significant level P = 0.10 with a minimum of five cases in each category is shown in italics below.

### 3.1. *Usual Occupation* (Table 4)

In the usual occupation category, significant excess risk in the major occupation groups (two-digit codes) was only observed at P = 0.10 for occupations in social sciences and related (SOC 23). Excess risks for minor groups (three-digit codes) were significant at P = 0.05 for occupations in fabricating, assembling and repairing wood products (SOC 854) and at P = 0.10 for occupations in other managers and administrators (SOC 113/114), bookkeeping and account-recording (SOC 413), and lodging and other accommodation services (SOC 613). Several unit occupations (four-digit codes) had significantly increased ORs at P = 0.05 for: *insurance sales jobs* (SOC 5171), *rail transport equipment mechanics and repairers* (SOC 8583), brick and stone masons-tile setters (SOC 8782), ship engineering officers (SOC 9153), and at P = 0.10 for: administrators in teaching and related fields (SOC 1133), financial management (SOC 1135), livestock farm workers (SOC 7183), sawmill sawyers and related (SOC 8231), cabinet and wood furniture makers (SOC 8541), and *taxi drivers and chauffeurs* (SOC 9173); the ORs were significantly low at P = 0.10 for sales clerks and salespersons in commodities, not else classified (SOC 5135) and welding and flame cutting (SOC 8335).

### 3.2. *Usual Industry* (Table 5)

In the usual industry category, a significant excess risk in the major industry group was noted at P = 0.05 for motor vehicle, parts and accessories, wholesale (SIC 55) and insurance and real estate agencies (SIC 76) and at P = 0.10 for beverage (SIC 11), federal government services (SIC 81) and *membership organizations* (SIC 98); ORs for printing, publishing and allied (SIC 28) industry was low at P = 0.10. Excess risks were significant at P = 0.05 for the following minor industry groups (three-digit codes): *brewery products* (SIC 113), defense services (SIC 811), and *human resource administration* (SIC 826) and at P = 0.10 for livestock farms (except animal specialties) (SIC 011), and dairy products (SIC 104). Odds ratio was significantly low at P = 0.10 for interior and finishing work (SIC 427). Several industry classes (four-digit codes) had significantly increased ORs at P = 0.05 for poultry and egg farms (SIC 0114), construction, mining machinery, materials handling (SIC 3192), exterior close-in: masonry work (SIC 4231), used goods moving and storage (SIC 4562), and specialty food stores (SIC 6012). ORs were significantly low at P = 0.10 for general freight trucking (SIC 4561), motor vehicle repair garages (general repairs) (SIC 6351) and general hospitals (SIC 8611).

In the validation study of the questionnaire, company job records of 81 individuals who reported having been employed in one of two large companies in BC were compared with information from their questionnaire; and the interclass correlation was 0.996 for starting year of employment and 0.971 for duration of employment.

## 4. Discussion

Significant associations between colon cancer and a number of occupations and industries were observed in our study. Several sedentary occupations in management, administration, bookkeeping and recording, financial management, insurance and real estate, and lodging and accommodation showed significantly increased risk of colon cancer either at P = 0.05 or at P = 0.10, which are consistent with findings from the previous studies [25–29].

In this study, we found some occupations and industries with significantly elevated risks of colon cancer in both ever and usual employment categories: workers working in occupations or industries with low physical activity, including insurance sales, human resource administration and membership organizations; rail transport equipment mechanics and repairers who were exposed to asbestos that has been reported to be associated with a higher risk of colon cancer; taxi drivers/chauffeurs who were exposed to engine exhaust and diesel engine emissions, a known carcinogen for colon cancer [21]; workers working in brewery products with exposure to chemicals such as ammonia refrigerant. Ammonia, potentially toxic to cells, shortens cell life span and alters DNA synthesis in various tissues including the ileum and colon [39] and is also thought to promote colon carcinogenesis in rats [40].

Elevated colon risks were also found in usual employment categories for occupations in wood products fabricating, assembling and repairing with exposure to wood dusts, a controversial agent without sufficient evidence of the association by the International Agency of Research on Cancer by far; industries of poultry and egg farms with exposure to gaseous agents such as ammonia from litter, carbon monoxide from poorly ventilated gas-fired heaters and hydrogen sulphide from liquid manure. Also, particles of organic or agricultural dust are aerosolized from poultry house litter; livestock farms (except animal specialties) and dairy products and relevant occupations with exposure to noxious gases such as hydrogen sulfide, and ammonia and methane in the ambient barn air; sawmill sawyers and workers working in motor vehicle parts and accessory wholesale and truck transportation on used goods, with exposure to engine exhaust and diesel engine emissions [21]; workers in construction, mining machinery material handling, brick and stone masons-tile setters as well as exterior close-in masonry work, with exposure to asbestos, which was widely used prior to 1978 in many building projects to increase the strength of concrete; ship engineering officers who were exposed to exhaust gases, engine emissions and asbestos; and workers in beverage industry with exposure to chemicals in gases and vapors typically involving chemical-handling activities related to cleaning operations, disinfection of process areas and use of preservatives in long-term food storage, in addition to thermal oils in the maintenance of heating and ammonia in cooling systems.

The strengths of this study are several folds. It is population-based with ascertainment of pathology confirmed cancer incident cases and includes lifetime occupational history with the ability to control for potential confounding factors and effect modifiers. Our study has revealed a number of occupational risk factors for colon cancer. Interpretation of those findings is limited by the lack of information on occupational exposures and the possibility that statistically significant results may have occurred by chance because of multiple comparisons; also, especially for many usual occupations and industries, numbers are few. In addition, other cancer cases used as controls may not be representative of the general population. Particularly, other cancers may also have some occupational risks that are common to those of colon cancer cases; in such situation, the corresponding estimated OR’s noted in this study would underestimate the true risks. A missing value category was used in the analysis to avoid reducing the study sample size and that may yield biased results. However, since the proportion of missing data is small and it is unlikely that missing information on education and smoking is correlated with job classification, the biases if any should be relatively small. Some of the confounding factors such as measures of body-mass index and physical activities were not collected for this study. Nevertheless, our validation study demonstrates that self-reported employment history bear little recall errors in out study.

Some of the risks observed may not apply to current workers because of a potential decrease in industrial exposures. However, the results of our study are in line such as elevated colon cancer risk by exposure to asbestos and to engine exhaust and diesel engine emissions from the literature and further suggest that exposure to wood dusts and to ammonia may carry an increased risk of colon cancer. More specific studies using population controls are needed to investigate associations between occupation and exposures to chemical substances, taking into account changes in concentration levels over time.

## Figures and Tables

**Table 1:** Cancer site distribution of 7,552 controls.

Site	Controls	Percent
lip	99	1.3%
oral cavity	390	5.2%
esophagus	174	2.3%
stomach	343	4.5%
liver	38	0.5%
pancreas	136	1.8%
larynx	280	3.7%
soft tissue sarcoma	101	1.3%
melanoma	458	6.1%
non-melanoma skin	1,050	13.9%
prostate	1,366	18.1%
testis	82	1.1%
bladder	972	12.9%
kidney	314	4.2%
brain	159	2.1%
Hodgkin’s Disease	52	0.7%
non-Hodgkin’s lymphoma	417	5.5%
multiple myeloma	118	1.6%
leukemia	184	2.4%
other sites	326	4.3%
multiple sites	493	6.5%
Total	7,552	100.0%

**Table 2:** Characteristics of cases and controls.

Colon Cancer Characteristics	Cases (*n* = 1,155)	Controls (*n* = 7,552)

	No. (%)	No. (%)
Year of diagnosis		
1983	218(18.9)	2,674(35.4)
1984	254(22.0)	1,660(22.0)
1985	259(22.4)	1,302(17.2)
1986	246(21.3)	1,010(13.4)
1987	178(15.4)	906(12.0)
Marital Status		
Single	56(4.8)	352(4.7)
Married/common law	958(82.9)	6,269(83.0)
Widowed	74(6.4)	463(6.1)
Separated/divorced	54(4.7)	405(5.4)
Not answered	13(1.1)	63(0.8)
Education		
≤7 years	136(11.8)	842(11.1)
8–11 years	511(44.2)	3,336(44.2)
High school	138(11.9)	830(11.0)
Post-secondary	329(28.5)	2,144(28.4)
Not answered	41(3.5)	400(5.3)
Alcohol consumption status		
Never drinker	137(11.9)	738(9.8)
Ever drinker	890(77.1)	5,456(72.2)
Not answered	128(11.1)	1,358(18.0)
Smoking duration, years		
0	282(24.4)	1,501(19.9)
1–29	379(32.8)	2,026(26.8)
30–44	284(24.6)	2,216(29.3)
45+	196(17.0)	1,682(22.3)
Not answered	14(1.2)	127(1.7)
Cigarette Pack-Years		
0	282(24.4)	1,501(19.9)
1–24	348(30.1)	2,039(27.0)
25–49	271(23.5)	2,012(26.6)
≥50	200(17.3)	1,582(20.9)
Unknown	54(4.7)	418(5.5)
Pipe smoking status		
Non-Pipe Smoker	1,098(95.1)	7,053(93.4)
Pipe Smoker	57(4.9)	499(6.6)
Respondent to questionnaire		
Patient	930(80.5)	5,955(78.9)
Proxy	199(17.2)	1,403(18.6)
Unknown	26(2.3)	194(2.6)

**Table 3:** Odds Ratios (OR) for potentially important confounding variables/effect modifiers.

Confounding Variable	Cases	Controls	OR	P-value	95% Confidence Interval (95% CI)
Smoking duration, years					
0	282	1,504	1.00		
1–29	379	2,026	1.05	0.60	0.87–1.26
30–44	284	2,220	0.69	<0.01	0.57–0.83
45+	196	1,684	0.57	<0.01	0.46–0.70
Not answered	14	118	0.58	0.06	0.33–1.02
Education					
≤7 years	136	843	1.00		
8–11 years	511	3,339	0.99	0.92	0.79–1.24
High school	138	830	1.05	0.72	0.80–1.37
Post-secondary	329	2,146	0.98	0.86	0.78–1.23
Not answered	41	394	0.65	0.03	0.44–0.96

**Table 4:** Odds Ratios for ever and usual occupations.

Code	Occupation Title	Ever					Usual				
		
		Case	OR	*P* value	95% CI		Case	OR	*P* value	95% CI	
11	Managerial, Administrative and Related	309	1.12	0.163	0.96	1.31	173	1.17	0.122	0.96	1.43
111	Officials and Administrators Unique to Government	40	1.19	0.343	0.83	1.71	17	1.18	0.548	0.69	2.03
1113	Administrators	11	1.35	0.390	0.68	2.68	5	1.37	0.539	0.50	3.74
1116	Inspectors and Regulatory Officers	21	1.17	0.524	0.72	1.90	11	1.33	0.402	0.68	2.59
113/114	Other Managers and Administrators	157	1.13	0.218	0.93	1.37	84	1.24	0.099	0.97	1.58
1130	General Managers and Other Senior Officials	62	1.26	0.130	0.93	1.70	28	1.30	0.226	0.85	1.99
1131	Management, Natural Sciences Engineering	7	0.75	0.486	0.33	1.69	6	1.27	0.616	0.50	3.23
1133	Administrators in Teaching and Related Fields	17	1.32	0.325	0.76	2.29	10	1.96	0.076	0.93	4.12
1135	Financial Management	27	1.33	0.190	0.87	2.04	17	1.59	0.099	0.93	2.73
1137	Sales and Advertising Management	54	0.92	0.597	0.68	1.25	20	0.79	0.331	0.49	1.29
1143	Production Management	31	0.94	0.764	0.63	1.41	11	0.96	0.904	0.49	1.86
1145	Management, Construction Operations	9	0.57	0.109	0.29	1.13	6	0.93	0.873	0.38	2.27
1147	Management, Transport, Communication Operations	25	1.43	0.120	0.91	2.24	9	1.05	0.895	0.51	2.17
1149	Other Managers and Administrators, NEC	48	1.26	0.178	0.90	1.76	17	1.41	0.208	0.83	2.41
117	Occupations Related to Management/Administration	68	1.10	0.504	0.83	1.46	25	0.87	0.538	0.56	1.36
1171	Accountants, Auditors and Other Financial Officers	46	1.15	0.410	0.83	1.60	23	1.02	0.934	0.64	1.63
21	Natural Sciences, Engineering and Mathematics	84	0.94	0.632	0.73	1.21	35	0.79	0.210	0.55	1.14
214/215	Architects, Engineers and Community Planners	25	0.90	0.642	0.58	1.40	13	0.95	0.869	0.52	1.75
216	Other Occupations in Architecture and Engineering	40	1.11	0.555	0.79	1.57	11	0.91	0.781	0.47	1.77
23	Social Sciences and Related	19	1.23	0.429	0.74	2.05	14	1.74	0.077	0.94	3.21
234	Law and Jurisprudence	10	1.47	0.298	0.71	3.04	8	1.56	0.282	0.69	3.51
2343	Lawyers and Notaries	10	1.88	0.095	0.90	3.94	7	1.72	0.221	0.72	4.10
25	Religion	11	1.05	0.886	0.54	2.05	9	1.30	0.505	0.60	2.81
2511	Ministers of Religion	11	1.10	0.785	0.56	2.18	9	1.30	0.505	0.60	2.81
27	Teaching and Related	52	0.88	0.441	0.64	1.22	24	0.79	0.308	0.50	1.24
271	University Teaching and Related	12	1.05	0.879	0.56	1.97	8	1.08	0.847	0.49	2.36
2711	University Teachers	10	0.99	0.977	0.50	1.95	8	1.18	0.680	0.54	2.59
273	Elementary and Secondary School Teaching	28	0.78	0.242	0.51	1.18	13	0.70	0.251	0.38	1.29
2733	Secondary School Teachers	14	0.81	0.473	0.46	1.44	6	0.69	0.408	0.29	1.66
31	Medicine and Health	31	0.81	0.299	0.54	1.21	22	0.83	0.436	0.52	1.33
311	Health Diagnosing and Treating	16	1.19	0.535	0.69	2.06	16	1.24	0.458	0.70	2,19
3111	Physicians and Surgeons	12	1.41	0.297	0.74	2.69	12	1.19	0.286	0.86	1.64
33	Artistic, Literary, Recreational and Related	36	1.02	0.916	0.71	1.47	15	1.36	0.294	0.77	2.42
331	Fine and Commercial Art, Photography and Related	9	0.80	0.539	0.39	1.63	8	1.53	0.290	0.70	3.36
41	Clerical and Related	195	1.00	0.912	0.85	1.21	54	0.99	0.948	0.73	1.34
413	Bookkeeping, Account-recording	65	1.08	0.597	0.81	1.44	20	1.66	0.052	1.00	2.77
4130	Supervisors	10	1.09	0.807	0.55	2.18	5	2.33	0.125	0.79	6.87
4131	Bookkeepers and Accounting Clerks	42	1.19	0.325	0.84	1.68	5	0.86	0.761	0.33	2.28
415	Material Recording, Scheduling and Distributing	61	0.78	0.075	0.59	0.03	11	0.67	0.223	0.35	1.28
4155	Stock Clerks	22	0.64	0.065	0.40	1.03	6	0.95	0.910	0.39	2.31
417	Reception, Information, Mail, Message Distribution	28	0.90	0.611	0.60	1.35	8	0.72	0.398	0.34	1.54
419	Other Clerical and Related	73	1.25	0.100	1.00	1.61	13	0.97	0.920	0.54	1.75
51	Sales	279	1.09	0.264	0.94	1.27	104	0.94	0.585	0.75	1.17
513/514	Sales, Commodities	196	0.98	0.815	0.83	1.16	70	0.81	0.115	0.62	1.05
5130	Supervisors	85	0.85	0.169	0.67	1.07	35	0.85	0.397	0.58	1.24
5133	Commercial Travellers	49	1.25	0.181	0.90	1.73	15	1.30	0.374	0.73	2.32
5135	Sales Clerks and Salespersons, Commodities, NEC	89	0.86	0.196	0.68	1.08	17	0.64	0.099	0.38	1.09
517	Sales, Services	72	1.39	0.021	1.05	1.84	24	1.29	0.273	0.82	2.04
5171	Insurance Sales	31	1.81	0.006	1.19	2.76	9	2.22	0.043	1.02	4.81
5172	Real Estate Sales	26	1.13	0.574	0.74	1.73	9	1.65	0.190	0.78	3.49
519	Other Sales	26	0.88	0.548	0.58	1.34	8	1.19	0.669	0.54	2.64
5193	Route Drivers	20	1.02	0.936	0.63	1.65	6	1.21	0.684	0.48	3.03
61	Services	365	0.96	0.581	0.83	1.11	79	0.95	0.688	0.74	1.22
611	Protective Services	260	0.98	0.815	0.83	1.16	35	0.89	0.542	0.61	1.29
6111	Firefighters	9	0.95	0.889	0.40	2.25	7	1.14	0.756	0.50	2.60
6112	Police Officers and Detectives, Government	16	1.03	0.913	0.61	1.75	6	0.79	0.596	0.33	1.89
6116	Commissioned Officers, Armed Forces	44	0.88	0.465	0.63	1.24	8	0.94	0.876	0.43	2.04
6117	Other Ranks, Armed Forces	195	1.06	0.530	0.88	1.27	7	0.76	0.499	0.349	1.68
612	Food and Beverage Preparation and Related	55	0.91	0.532	0.68	1.22	11	0.67	0.202	0.36	1.24
6121	Chefs and Cooks	24	1.06	0.797	0.68	1.65	5	0.70	0.454	0.28	1.78
613	Lodging and Other Accommodation Services	28	0.82	0.346	0.54	1.24	8	2.00	0.099	0.89	4.56
6130	Supervisors	23	0.81	0.371	0.51	1.29	8	1.90	0.128	0.83	4.34
619	Other Service	63	1.12	0.440	0.84	1.49	18	1.09	0.749	0.64	1.85
6190	Supervisors	13	2.01	0.037	1.04	3.87	5	2.05	0.180	0.72	5.85
6191	Janitors, Charworkers and Cleaners	46	1.09	0.603	0.79	1.51	11	0.81	0.524	0.42	1.55
71	Farming, Horticultural and Animal Husbandry	308	0.92	0.282	0.79	1.07	95	1.06	0.647	0.83	1.36
711	Farmers	182	1.00	0.919	0.83	1.22	79	1.13	0.353	0.87	1.46
7113	Livestock Farmers	46	1.22	0.247	0.87	1.71	19	1.30	0.323	0.77	2.19
7115	Crop Farmers	46	1.09	0.619	0.78	1.53	16	0.96	0.886	0.55	1.68
718/719	Other Farming, Horticulture and Animal Husbandry	63	0.84	0.234	0.63	1.12	6	1.02	0.965	0.43	2.44
7183	Livestock Farm Workers	31	1.00	0.961	0.68	1.51	5	2.58	0.077	0.90	7.36
7195	Nursery and Related Workers	21	1.19	0.485	0.73	1.94	6	1.47	0.420	0.58	3.75
7199	Other Farming, Horticulture and Animal Husbandry, NEC	80	0.86	0.228	0.67	1.10	7	0.88	0.754	0.40	1.96
73	Fishing, Trapping and Related	47	1.20	0.278	0.86	1.67	13	0.93	0.814	0.51	1.70
7313	Net, Trap and Line Fishing	44	1.25	0.198	0.89	1.76	12	0.91	0.766	0.49	1.70
75	Forestry and Logging	121	1.01	0.924	0.82	1.24	31	1.30	0.198	0.87	1.94
7510	Foremen/women	21	1.09	0.724	0.68	1.76	6	1.14	0.778	0.46	2.84
7513	Timber Cutting and Related	64	1.07	0.646	0.80	1.43	13	1.39	0.294	0.75	2.57
77	Mining and Quarrying	71	0.88	0.327	0.68	1.14	16	1.36	0.294	0.77	2.42
7717	Mineral Cutting, Handling and Loading	34	0.79	0.235	0.54	1.17	8	1.40	0.405	0.63	3.09
81/82	Materials Processing	54	0.78	0.096	0.58	1.05	74	1.02	0.881	0.79	1.32
813/814	Metal Processing and Related	18	0.82	0.435	0.50	1.35	5	0.96	0.933	0.37	2.50
821/822	Food, Beverage and Related Processing	47	1.31	0.113	0.94	1.83	12	1.03	0.926	0.55	1.92
8215	Slaughtering, Meat Cutting, Canning, Curing, Packing	25	1.29	0.259	0.83	2.01	11	1.55	0.221	0.77	3.13
823	Wood Processing (Non-Pulp and Paper Production)	132	1.13	0.247	0.92	1.39	41	1.28	0.173	0.90	1.83
8231	Sawmill Sawyers and Related	43	1.19	0.306	0.85	1.66	16	1.77	0.053	0.99	3.15
8238	Labouring	51	1.11	0.520	0.81	1.53	7	0.93	0.861	0.41	2.10
8239	Wood Processing, Non-Pulp and Paper, NEC	40	1.23	0.241	0.87	1.74	8	1.50	0.327	0.67	3.37
825	Pulp and Paper Production and Related	21	0.85	0.504	0.53	1.37	5	0.74	0.524	0.29	1.87
83	Machining and Related	117	1.05	0.650	0.85	1.30	31	0.73	0.106	0.50	1.07
831	Metal Machining	51	1.27	0.145	0.92	1.75	15	1.09	0.771	0.61	1.95
8313	Machinist and Machine Tool Setting-up	37	1.14	0.497	0.78	1.66	6	0.58	0.219	0.24	1.38
833	Metal Shaping and Forming, Except Machining	62	1.08	0.597	0.81	1.44	15	0.71	0.217	0.41	1.22
8335	Welding and Flame Cutting	36	0.99	0.957	0.69	1.42	7	0.49	0.081	0.22	1.09
85	Product Fabricating, Assembling and Repairing	274	1.05	0.508	0.91	1.21	114	1.08	0.487	0.87	1.34
853	Electrical, Electronic Equipment	56	1.36	0.506	1.00	1.85	13	0.69	0.219	0.38	1.25
8533	Electrical Equipment Installing, Repairing, NEC	33	1.38	0.111	0.93	2.05	8	0.80	0.554	0.38	1.68
854	Fabricating, Assembling and Repairing: Wood Products	23	1.30	0.269	0.82	2.07	9	2.29	0.045	1.02	5.15
8541	Cabinet and Wood Furniture Makers	15	1.48	0.195	0.82	2.68	6	2.51	0.063	0.95	6.64
858	Mechanics and Repairers, NEC	159	1.10	0.319	0.91	1.33	69	1.11	0.437	0.85	1.44
8581	Motor Vehicle Mechanics and Repairers	63	1.08	0.597	0.81	1.44	20	0.87	0.572	0.54	1.41
8582	Aircraft Mechanics and Repairers	24	1.35	0.211	0.84	2.16	5	1.15	0.783	0.43	3.10
8583	Rail Transport Equipment Mechanics and Repairers	11	3.84	<0.001	1.82	8.11	6	6.06	0.001	2.04	18.00
8584	Other Industrial Equipment Repairers	64	0.96	0.774	0.73	1.27	28	1.03	0.888	0.68	1.55
8589	Other Mechanics and Repairers, NEC	13	0.98	0.948	0.54	1.79	6	2.02	0.147	0.78	5.22
859	Other Product Fabrication, Assembling, Repairing	49	0.93	0.658	0.67	1.28	11	1.17	0.643	0.60	2.27
8592	Marine Craft Fabricating, Assembling, Repairing	36	1.01	0.958	0.70	1.46	9	1.65	0.198	0.77	3.54
87	Construction Trades	273	0.90	0.184	0.77	1.05	110	0.85	0.137	0.69	1.05
871	Excavating, Grading, Paving	63	0.94	0.671	0.71	1.25	15	0.66	0.130	0.39	1.13
8711	Excavating, Grading and Related	31	1.02	0.922	0.69	1.52	9	0.80	0.539	0.39	1.63
873	Electrical, Wire Communications Installing/Repair	42	1.03	0.867	0.73	1.46	18	1.00	0.999	0.60	1.71
8733	Electricians and Repairers	24	1.07	0.771	0.68	1.69	12	1.37	0.336	0.72	2.60
878/879	Other Construction Trade	112	0.84	0.116	0.68	1.04	50	0.85	0.319	0.62	1.17
8780	Foremen/women	20	0.92	0.728	0.58	1.47	6	0.96	0.927	0.40	2.31
8781	Carpenters and Related	70	0.86	0.260	0.66	1.12	27	0.80	0.303	0.52	1.22
8782	Brick and Stone Masons, Tile Setters	9	1.29	0.493	0.62	2.67	7	2.97	0.018	1.20	7.34
8791	Pipefitting, Plumbing and Related	27	0.99	0.964	0.64	1.52	17	1.20	0.499	0.71	2.04
8798	Labouring	44	0.94	0.717	0.67	1.31	5	0.75	0.550	0.29	1.93
8799	Other Construction Trade, NEC	10	0.80	0.507	0.41	1.55	5	1.79	0.256	0.66	4.89
91	Transport Equipment Operating	246	1.15	0.077	0.99	1.34	86	1.15	0.279	0.89	1.48
911	Air Transport	24	1.51	0.084	0.95	2.41	10	1.39	0.359	0.69	2.81
9111	Air Pilots, Navigators and Flight Engineers	16	1.29	0.368	0.74	2.25	7	1.24	0.618	0.53	2.89
913	Railway Transport	23	1.14	0.570	0.73	1.79	12	1.29	0.440	0.68	2.46
9131	Locomotive Operating	8	0.71	0.387	0.33	1.54	6	1.40	0.480	0.55	3.56
9133	Conductors and Brake Workers, Railway	13	1.79	0.073	0.95	3.38	6	2.05	0.146	0.78	5.40
915	Water Transport	51	1.17	0.325	0.86	1.60	16	1.17	0.573	0.68	2.02
9151	Deck Officers	13	0.88	0.678	0.48	1.61	6	0.94	0.890	0.39	2.26
9153	Engineering Officers, Ship	10	1.56	0.219	0.77	3.17	7	4.80	0.002	1.81	12.71
917	Motor Transport, Other Transport Equipment	157	1.10	0.319	0.91	1.33	46	1.01	0.952	0.73	1.40
9171	Bus Drivers	20	0.84	0.479	0.52	1.36	7	0.80	0.593	0.36	1.77
9173	Taxi Drivers and Chauffeurs	30	1.54	0.045	1.01	2.25	6	2.25	0.088	0.89	5.71
9175	Truck Drivers	124	1.08	0.460	0.88	1.33	32	0.93	0.716	0.63	1.38
93	Material Handling and Related, NEC	101	1.04	0.734	0.83	1.30	26	0.99	0.963	0.64	1.52
9313	Longshore Workers, Stevedores and Freight Handlers	48	1.13	0.462	0.82	1.57	16	1.33	0.320	0.76	2.33
9315	Material Handling Equipment Operators, NEC	21	0.93	0.759	0.59	1.48	5	0.94	0.896	0.37	2.39
95	Other Crafts and Equipment Operating	68	1.11	0.460	0.84	1.46	29	1.02	0.925	0.67	1.55
953	Stationary Engine, Utilities Equipment Operating	37	1.16	0.432	0.80 5	1.68	16	1.22	0.477	0.71	2.11
9539	Stationary Engine, Utility Equipment, NEC	25	1.03	0.892	0.67	1.58	12	1.18	0.612	0.62	2.24
955	Electronic Communications Equipment, NEC	19	1.46	0.158	0.86	2.47	9	1.75	0.159	0.80	3.82

**Table 5:** Odds Ratios for ever and usual industries.

Code	Industry Title	Ever					Usual				
		
		Case	OR	*P* value	95% CI		Case	OR	*P* value	95% CI	
01	Agriculture	296	0.90	0.184	0.77	1.05	87	1.00	0.999	0.78	1.31
011	Livestock Farms (except Animal Specialities)	72	1.13	0.377	0.86	1.48	22	1.53	0.085	0.94	2.48
0111	Dairy Farms	49	1.39	0.046	1.01	1.92	8	1.13	0.757	0.52	2.45
0112	Cattle Farms	13	0.88	0.678	0.48	1.61	6	1.33	0.556	0.52	3.43
0114	Poultry and Egg Farms	17	1.56	0.116	0.90	2.72	6	3.52	0.016	1.26	9.81
013	Field Crop Farms	49	0.96	0.806	0.69	1.33	9	0.98	0.958	0.46	2.09
0131	Wheat Farms	34	0.95	0.792	0.65	1.39	6	0.92	0.861	0.36	2.34
015	Fruit and Other Vegetable Farms	35	1.04	0.837	0.72	1.51	9	0.95	0.889	0.46	1.95
0151	Fruit Farms	32	1.04	0.844	0.71	1.54	9	1.07	0.856	0.52	2.22
017	Livestock, Field Crop, Horticultural Combination Farms	179	0.87	0.157	0.72	1.06	45	0.93	0.674	0.66	1.31
03	Fishing and Trapping	49	1.08	0.631	0.79	1.48	14	0.90	0.725	0.50	1.62
031	Fishing	46	1.15	0.410	0.83	1.60	13	0.91	0.758	0.50	1.66
0311	Salt Water Fishing	42	1.14	0.463	0.80	1.61	12	0.87	0.656	0.47	1.61
04	Logging	135	0.99	0.920	0.81	1.20	38	1.13	0.515	0.78	1.63
06	Mining	95	0.87	0.260	0.68	1.11	21	0.89	0.627	0.56	1.42
061	Metal Mines	71	0.83	0.181	0.63	1.09	17	0.90	0.693	0.53	1.52
0614	Silver-Lead-Zinc Mines	23	1.10	0.682	0.70	1.74	5	0.95	0.920	0.35	2.59
0631	Bituminous Coal Mines	28	1.08	0.719	0.71	1.64	5	1.25	0.662	0.46	3.40
10	Food	86	1.08	0.537	0.85	1.38	22	0.96	0.863	0.60	1.53
101	Meat and Poultry Products	18	0.92	0.748	0.55	1.53	5	0.73	0.514	0.28	1.88
1011	Meat and Meat Products (Except Poultry)	18	1.01	0.970	0.60	1.71	5	0.77	0.599	0.29	2.04
104	Dairy Products	21	1.34	0.239	0.82	2.18	8	2.02	0.091	0.90	4.56
1041	Fluid Milk	13	1.45	0.242	0.78	2.70	5	2.08	0.173	0.73	5.96
11	Beverage	13	1.24	0.482	0.68	2.26	6	2.25	0.084	0.90	5.64
113	Brewery Products	8	1.99	0.090	0.90	4.41	5	3.71	0.015	1.29	10.66
25	Wood	189	1.07	0.461	0.89	1.28	67	1.10	0.504	0.83	1.46
251	Sawmill, Planing Mill and Shingle Mill Products	153	1.02	0.838	0.84	1.23	56	1.15	0.370	0.85	1.56
2512	Sawmill, Planing Mill (Except Shingles and Shakes)	147	1.03	0.773	0.84	1.26	54	1.18	0.295	0.87	1.61
252	Veneer and Plywood	22	1.25	0.356	0.78	2.01	9	1.18	0.656	0.57	2.45
2522	Softwood Veneer and Plywood	20	1.43	0.161	0.87	2.36	9	1.55	0.248	0.74	3.26
26	Furniture and Fixtures	16	1.14	0.639	0.66	1.97	6	1.52	0.366	0.61	3.77
261	Household Furniture	14	1.19	0.569	0.65	2.17	5	1.46	0.450	0.55	3.907
27	Paper and Allied Products	49	0.89	0.451	0.66	1.21	19	0.81	0.393	0.50	1.31
271	Pulp and Paper Production	45	0.87	0.407	0.63	1.21	18	0.80	0.394	0.48	1.34
2711	Pulp Industry	35	0.79	0.210	0.55	1.14	16	0.87	0.617	0.50	1.50
28	Printing, Publishing and Allied	24	0.81	0.348	0.52	1.26	8	0.52	0.082	0.25	1.09
281	Commercial Printing	10	0.93	0.832	0.48	1.82	5	0.89	0.811	0.34	2.31
29	Primary Metal	48	0.77	0.106	0.56	1.06	16	0.78	0.358	0.46	1.33
299	Other Rolled, Cast, Extruded Non Ferrous Metal Products	24	0.92	0.717	0.59	1.45	10	0.98	0.954	0.49	1.95
30	Fabricated Metal Products(Non Machinery, Transport)	69	1.13	0.377	0.86	1.48	19	0.92	0.748	0.55	1.53
308	Machine Shops	28	1.49	0.068	0.97	2.29	6	1.18	0.715	0.49	2.87
31	Machinery (except Electrical)	28	1.63	0.025	1.07	2.50	8	1.57	0.262	0.71	3.46
319	Other Machinery and Equipment	24	1.82	0.012	1.14	2.90	8	1.86	0.135	0.83	4.20
3192	Construction, Mining Machinery, Materials Handling	7	1.88	0.146	0.80	4.41	5	3.66	0.023	1.19	11.22
32	Transportation Equipment	115	0.98	0.852	0.79	1.21	19	0.93	0.777	0.56	1.54
327	Shipbuilding and Repairs	73	0.95	0.705	0.73	1.24	12	1.01	0.975	0.54	1.88
33	Electrical and Electronic Products	21	1.23	0.401	0.76	1.99	6	0.95	0.907	0.40	2.25
36	Refined Petroleum and Coal Products	16	1.29	0.368	0.74	2.24	7	1.61	0.288	0.67	3.88
361	Refined Petroleum Products	13	1.17	0.615	0.63	2.16	7	1.61	0.297	0.66	3.94
3611	Refined Petroleum Products(Non Lubricating Oil, Grease)	13	1.17	0.615	0.63	2.16	7	1.61	0.297	0.66	3.94
37	Chemical and Chemical Products	31	1.42	0.091	0.95	2.13	10	1.15	0.695	0.57	2.31
40	Building, Developing and General Contracting	130	0.93	0.497	0.75	1.15	42	0.94	0.717	0.67	1.31
401	Residential Building and Development	29	0.93	0.728	0.62	1.40	8	1.00	0.999	0.46	2.23
4011	Single Family Housing	26	1.00	0.964	0.65	1.56	8	1.25	0.583	0.56	2.78
41	Industrial and Heavy Construction	53	0.78	0.096	0.58	1.05	15	0.72	0.228	0.42	1.23
412	Highway and Heavy Construction	44	0.82	0.243	0.59	1.14	13	0.78	0.400	0.44	1.39
4121	Highways, Streets and Bridges	29	0.93	0.728	0.62	1.40	9	0.99	0.978	0.48	2.39
42	Trade Contracting	109	0.90	0.342	0.72	1.12	48	0.90	0.514	0.66	1.23
422	Structural and Related Work	20	0.90	0.669	0.56	1.46	6	0.73	0.481	0.30	1.75
423	Exterior Close-In Work	14	1.18	0.582	0.66	2.13	8	1.79	0.150	0.81	3.96
4231	Exterior Close-In: Masonry Work	5	1.10	0.846	0.42	2.88	5	3.02	0.045	1.03	8.89
424	Plumbing, Heating, Air Conditioning Mechanical	23	1.18	0.484	0.74	1.88	12	1.07	0.834	0.57	2.01
4241	Plumbing	13	0.90	0.734	0.49	1.65	6	0.75	0.519	0.31	1.80
426	Electrical Work	23	1.26	0.334	0.79	2.01	10	1.48	0.285	0.72	3.04
427	Interior and Finishing Work	21	0.69	0.112	0.44	1.09	6	0.44	0.051	0.19	1.01
45	Transportation	282	1.21	0.011	1.05	1.40	106	1.08	0.513	0.86	1.36
451	Air Transport	24	1.26	0.320	0.80	1.99	12	1.07	0.834	0.57	2.01
4511	Scheduled Air Transport	16	0.96	0.882	0.56	1.65	10	1.03	0.933	0.52	2.04
453	Rail Transport and Related Service	78	1.10	0.459	0.86	1.42	35	1.17	0.419	0.80	1.71
4531	Railway Transport	77	1.10	0.459	0.86	1.42	34	1.16	0.449	0.79	1.70
454	Water Transport	54	1.36	0.051	1.00	1.85	9	0.70	0.316	0.35	1.41
4541	Freight and Passenger Water Transport	42	1.52	0.020	1.07	2.16	5	0.68	0.419	0.27	1.73
455	Services Incidental to Water Transport	25	1.46	0.109	0.92	2.32	11	1.33	0.413	0.67	2.63
4551	Marine Cargo Handling	15	1.24	0.458	0.70	2.19	10	1.54	0.232	0.76	3.13
456	Truck Transport	72	1.02	0.881	0.79	1.32	22	0.93	0.759	0.59	1.48
4561	General Freight Trucking	50	1.01	0.950	0.74	1.37	11	0.59	0.096	0.32	1.10
4562	Used Goods Moving and Storage	7	1.92	0.133	0.82	4.50	5	7.14	0.002	2.11	24.13
457	Public Passenger Transit Systems	26	0.91	0.659	0.60	1.38	8	0.83	0.629	0.39	1.77
4571	Urban Transit Systems	16	0.82	0.474	0.48	1.41	5	0.78	0.610	0.30	2.03
458	Other Transportation	28	1.58	0.038	1.03	2.43	5	1.90	0.209	0.70	5.17
4581	Taxicab	26	1.52	0.070	0.97	2.39	5	1.90	0.209	0.70	5.17
48	Communications	54	1.06	0.709	0.78	1.44	25	0.91	0.673	0.59	1.41
481	Telecommunication Broadcasting	9	1.30	0.486	0.62	2.72	5	1.55	0.398	0.56	4.29
4821	Telecommunication Carriers	26	1.33	0.203	0.86	2.06	13	1.16	0.629	0.64	2.12
484	Postal and Courier Services	20	0.79	0.354	0.48	1.30	7	0.53	0.122	0.24	1.18
4841	Postal Service	18	0.74	0.253	0.44	1.24	7	0.53	0.122	0.24	1.18
49	Other Utility	34	0.95	0.782	0.66	1.37	18	1.11	0.692	0.66	1.86
4911	Electric Power Systems	19	0.83	0.456	0.51	1.36	10	0.91	0.789	0.46	1.61
51	Petroleum Products, WH	15	0.87	0.631	0.49	1.54	6	0.74	0.492	0.31	1.75
521	Food, Beverage, Drug and Tobacco, WH	29	1.29	0.230	0.85	1.96	7	0.69	0.369	0.31	1.55
55	Motor Vehicle, Parts and Accessories, WH	15	1.40	0.256	0.78	2.50	7	2.92	0.019	1.20	7.13
56	Metal, Hardware, Plumbing, Heating, Building Material, WH	40	1.26	0.211	0.88	1.81	12	0.99	0.975	0.52	1.87
563	Lumber and Building Materials, WH	25	1.24	0.334	0.80	1.92	11	1.44	0.297	0.73	2.86
5631	Lumber, Plywood and Millwork, WH	15	1.37	0.278	0.78	2.42	5	1.23	0.679	0.46	3.28
57	Machinery, Equipment and Supplies, WH	45	1.23	0.223	0.88	1.72	19	1.24	0.406	0.75	2.06
579	Other Machinery, Equipment and Supplies, WH	12	0.99	0.975	0.53	1.83	5	1.26	0.639	0.48	3.31
59	Other Products, WH	25	0.87	0.519	0.57	1.33	6	0.68	0.380	0.29	1.61
60	Food, Beverage and Drugs, Retail	55	0.85	0.291	0.63	1.15	23	1.01	0.966	0.64	1.59
601	Food Stores	48	0.92	0.597	0.68	1.25	19	1.15	0.591	0.69	1.91
6011	Groceries Food Stores	29	0.79	0.260	0.52	1.19	8	0.68	0.319	0.32	1.45
6012	Specialty Food Stores	23	1.29	0.288	0.81	2.06	10	2.58	0.023	1.14	5.84
62	Household Furniture, Appliances, Furnishing, Retail	22	0.98	0.933	0.61	1.57	5	0.59	0.267	0.23	1.50
63	Auto Vehicle, Parts, Accessories, Sale and Service	86	0.95	0.688	0.74	1.22	28	0.81	0.324	0.53	1.23
631	Automobile Dealers	23	1.17	0.497	0.74	1.84	11	1.40	0.333	0.71	2.77
6311	Automobile (New) Dealers	23	1.25	0.341	0.79	1.98	11	1.52	0.227	0.77	3.00
635	Motor Vehicle Repair Shops	48	0.96	0.806	0.69	1.33	13	0.72	0.273	0.40	1.30
6351	Motor Vehicle Repair Garages (General Repairs)	42	0.97	0.860	0.69	1.36	8	0.50	0.064	0.24	1.04
64	General Retail Merchandising	43	0.83	0.266	0.60	1.15	16	0.90	0.704	0.52	1.55
641	General Merchandise Stores	43	0.83	0.266	0.60	1.15	16	0.90	0.704	0.52	1.55
6411	Department Stores	36	0.91	0.612	0.63	1.31	14	1.02	0.948	0.56	1.85
65	Other Retail Stores	36	0.93	0.703	0.64	1.35	9	0.64	0.202	0.32	1.27
70	Deposit Accepting Intermediaries	19	0.72	0.183	0.44	1.17	11	0.98	0.953	0.50	1.91
702	Chartered Banks, Other Banking-Type Intermediaries	14	0.64	0.118	0.37	1.12	8	0.92	0.828	0.43	1.95
7021	Chartered Banks	13	0.66	0.172	0.36	1.20	7	0.87	0.737	0.39	1.96
73	Insurance Underwriters	27	1.63	0.032	1.04	2.55	9	1.12	0.759	0.54	2.31
733	Property and Casualty Insurers	14	1.46	0.219	0.80	2.67	7	1.51	0.342	0.65	3.53
7339	Other Property and Casualty Insurers	13	1.38	0.309	0.74	2.57	6	1.25	0.633	0.50	3.12
76	Insurance and Real Estate Agencies	54	1.26	0.146	0.92	1.72	26	1.67	0.024	1.07	2.60
77	Business Services	79	1.09	0.508	0.85	1.41	30	1.03	0.888	0.68	1.55
773	Accounting and Bookkeeping Services	10	0.82	0.572	0.41	1.63	5	0.98	0.968	0.37	2.62
775	Architectural, Engineering and Other Services	37	1.17	0.402	0.81	1.69	14	0.97	0.920	0.54	1.75
7752	Offices of Engineers	20	1.20	0.473	0.73	1.98	6	0.75	0.519	0.31	1.80
7759	Other Scientific and Technical Services	16	1.14	0.639	0.66	1.97	6	1.21	0.684	0.48	3.03
81	Federal Government Services	348	1.12	0.131	0.97	1.30	86	1.24	0.099	0.97	1.58
811	Defence Services	316	1.11	0.168	0.96	1.29	66	1.41	0.018	1.06	1.88
815	General Administrative Service	17	1.00	0.971	0.59	1.74	6	0.70	0.417	0.30	1.66
817	Economic Service Administration	9	0.58	0.132	0.29	1.18	6	0.83	0.675	0.35	1.98
82	Provincial and Territorial Government Services	45	1.11	0.538	0.80	1.55	20	1.24	0.406	0.75	2.06
822	Protective Services	15	1.09	0.764	0.62	1.91	6	1.12	0.803	0.46	2.73
826	Human Resource Administration	10	2.34	0.024	1.11	4.89	7	4.72	0.002	1.79	12.46
83	Local Government Services	33	0.84	0.345	0.59	1.20	18	0.96	0.878	0.57	1.62
8323	Police Services	8	1.17	0.692	0.54	2.54	6	1.85	0.193	0.73	4.67
8324	Firefighting Services	9	1.50	0.289	0.71	2.17	7	1.83	0.168	0.78	4.32
85	Educational Services	88	1.05	0.704	0.82	1.35	48	1.01	0.952	0.73	1.40
851	Elementary and Secondary Education	63	1.08	0.597	0.81	1.44	33	1.02	0.919	0.70	1.50
853	University Education	21	1.17	0.524	0.72	1.90	11	1.07	0.843	0.55	2.09
86	Health and Social Services	55	0.98	0.896	0.72	1.33	28	0.98	0.925	0.65	1.49
861	Hospitals	24	0.75	0.196	0.49	1.16	9	0.64	0.224	0.31	1.31
8611	General Hospitals	18	0.68	0.124	0.42	1.11	6	0.47	0.082	0.20	1.10
865	Offices: Physicians, Surgeons and Dentists, Private	15	1.31	0.368	0.73	2.36	13	1.27	0.455	0.68	2.38
8651	Offices: Physicians, General Practice	7	1.48	0.371	0.63	3.49	6	1.57	0.341	0.62	3.98
91	Accommodation Services	48	0.91	0.554	0.67	1.24	15	1.39	0.259	0.78	2.46
911	Hotels, Motels and Tourist Courts	46	0.95	0.749	0.69	1.30	13	1.26	0.464	0.68	2.34
9111	Hotels and Motor Hotels	35	0.88	0.488	0.61	1.26	9	1.09	0.812	0.54	2.22
96	Amusement and Recreational Services	27	0.97	0.888	0.64	1.48	6	0.97	0.946	0.40	2.36
97	Personal and Household Services	19	0.85	0.522	0.52	1.40	9	0.93	0.843	0.46	1.90
98	Membership Organizations	39	1.57	0.017	1.08	2.28	16	1.78	0.058	0.98	3.23
981	Religious Organizations	15	1.17	0.595	0.66	2.09	8	1.20	0.658	0.54	2.69
99	Other Services	27	0.65	0.040	0.43	0.98	8	0.69	0.327	0.33	1.45
